# Local knowledge and practices towards malaria in an irrigated farming community in Ghana

**DOI:** 10.1186/s12936-018-2291-8

**Published:** 2018-04-04

**Authors:** Hector Attu, Jones K. Adjei

**Affiliations:** 1 Department of Geography & Resource Development, University of Ghana, Accra, Ghana; 2 Department of Humanities & Social Sciences, Red Deer College, Red Deer, AB Canada

**Keywords:** Malaria, Community perceptions, Irrigation agriculture, Ghana, Sub-Saharan Africa

## Abstract

**Background:**

Although malaria is endemic across Ghana, the risk is generally elevated for residents living in and around stagnant water bodies such as dams and irrigated farming projects. What knowledge do these at-risk populations have about the aetiology and symptoms of malaria? What are their coping strategies? And what interventions are needed to help improve the health outcomes of people living in high-risk malaria communities?

**Methods:**

This study addressed these research questions with primary data, comprising both qualitative interviews and quantitative surveys, collected in Asutsuare—a rural irrigated farming community located in the Greater Accra Region of Ghana.

**Results:**

Results from the fieldwork showed that awareness of malaria as a major health concern in the community was universal. Respondents also displayed a high knowledge of some common clinical symptoms of malaria. Yet, only 3% out of the total survey respondents of 337 indicated they immediately visit a health facility for treatment whenever they suspected malaria. The overwhelming majority (about 97%) indicated they only visit a healthcare facility for treatment if they felt the suspected malaria illness was severe and/or other treatment options had failed.

**Conclusion:**

Malaria testing training for drug dispensing personnel as well as the provision of malaria testing kits in drug dispensing stores are necessary to facilitate early malaria screening and timely diagnosis particularly in rural endemic areas.

## Background

Malaria is endemic in many tropical and sub-tropical areas of the world, with sub-Saharan Africa accounting for more than 90% of the global malaria cases [1]. Though there are regional and seasonal variations in prevalence rates, it is generally observed that reported malaria cases are fairly stable and often higher in and around irrigated farming areas compared with neighboring areas without irrigation agriculture [2–7]; for some exemptions, see for example, [8]. The often-high incidence of malaria in irrigated farming communities has largely been attributed to the availability of water from the irrigation system which provides a stable thriving environment for the mosquito vectors, resulting in high anopheline mosquito density [9, 10].

In spite of the reported malaria risks associated with the development of irrigation schemes, irrigation has nonetheless become an important strategy for food security, economic growth, and poverty alleviation for many developing countries like Ghana, a country where malaria has persistently remained a leading public health menace accounting for about 38% of all outpatient illnesses and almost half (48%) of all deaths in children under the age of 5 years old in the year 2015 [11].

As reported in other countries like India and Ethiopia, researchers in Ghana have also noted an increase in malaria prevalence following the construction of local irrigation projects. For example, a study by [12] in the Kassena-Nankana District in the Upper East Region of Ghana, which has one of the largest irrigation sites in the country, showed that malaria transmission was higher for people in the irrigated communities than in the non-irrigated ones. Another study by [13] observed a very high prevalence of malaria in the Sissala West District in the Upper West Region of Ghana, a situation they attributed to the presence of irrigation dams in the district. Also, [9, 14] in their study on the ecology of mosquitoes in an irrigated vegetable farm in Kumasi in the Ashanti Region of Ghana, argued that the irrigated farms had significantly multiplied the growth and survival of the mosquito larval and hence malaria transmission in the city.

However, to date, not much is known about the local knowledge about the aetiology and symptoms of malaria, practices and treatment options among residents in irrigated communities in Ghana. This neglect is unfortunate. As several authors have argued, the study of local peoples’ knowledge, attitudes as well as perceptions of malaria are important for the design and implementation of effective malaria control strategies [15–17].

This study, therefore, seeks to examine local knowledge and practices about malaria, and the expectations about malaria control, management, and interventions in an irrigated rural community in the Greater Accra Region of Ghana. Results from this study could contribute important insights to the collaborative nature of malaria control efforts among governmental, non-governmental, health agencies, agricultural and industrial sectors, and the general public.

## Methods

**Fig. 1:**
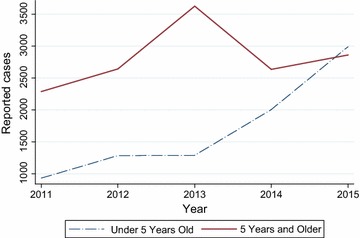
Cases of reported malaria per age group in Asutuare, 2011–2015

The study was conducted in Asutsuare, a town situated in the Shai-Osudoku District in the Greater Accra Region of Ghana. Fieldwork was conducted in February 2016. The study area has a population of about 2000 people, mostly belonging to the Ga-Dangbe ethnic group [18]. The main economic activity in the area is agriculture, which is facilitated by the Kpong Irrigation Project (K.I.P). Asutsuare was selected as the study area primarily because it is an irrigated community where stagnant water from the irrigated rice fields provides a natural condition for the proliferation of mosquitoes. Indeed, available evidence from the local District Health Directorate indicates that the Asutsuare area has consistently recorded the highest incidence of malaria cases in the entire Shai-Osudoku District over the past decade [19]. Information obtained during field research from the local District Health Directorate contained data on the reported cases of malaria in the study area between 2011 and 2015, disaggregated by age groups (under age 5 and 5 years and over). Actual population figures for the area over that time interval (2011 to 2015) were however not available. Figure 1 shows the available information used to present the trends in reported cases of malaria in the study area over a 5-year period.

As a rural farming community, it is likely that many residents spend most of their time working in the fields which exposes them to the malaria vectors. Also, as low-income earners, most of the residents may not have enough incomes to afford effective preventive strategies against malaria. It is against this backdrop that this study seeks to investigate how members in this community understand and cope with the issue of malaria.

The study utilized a mixed method approach. The first stage involved the administration of a survey using a stratified sampling technique based on the eight neighbourhoods of Asutsuare: SDA, Local Council, Gbese Dorm, Tsangme, Agave, Fada Korpe, Sikaman, and Estate. These eight neighbourhoods served as the strata from which individuals (the unit of analysis) were randomly selected for the study to improve the representativeness of our sample. A total of 337 adult individuals, about 42 from each neighbourhood were sampled, representing about 15% of the study population. The questionnaires took an average of 45 min to complete, with a completion rate of 95%. The questionnaire included items on demographic characteristics of the respondents (gender, age, level of education, employment status, religious affiliation, and ethnicity), their knowledge about the causes and prevention of malaria, and their coping strategies to malaria.

A bivariate analysis was undertaken to access whether respondents’ socio-demographic characteristics associated with their perceived causes of malaria. A Chi squared test of independence was initially used to tease out these associations but the results showed that much of the expected cell frequencies had a value less than 5, indicating that the results from the Chi squared tests were unstable [20]. All categories in the variables were recoded into binaries in order to compute the Fisher’s exact test of independence. However, some cell frequencies were too large for our computational programming to handle so this was utilized. In addition, the percent distributions were also used to further explore patterns and assess the extent of relationship between the variables.

The second component of this study involved a qualitative in-depth interview with six key informants comprising the local political representative of the area (popularly called an Assembly Member), the traditional Chief of Asutsuare, a health official of the Osudoku Health Centre, a field officer of the K.I.P, a leader of farmers connected to the K.I.P, and a local drug store operator. All interviews conducted were audio recorded with permission from interviewees. The audiotapes were then transcribed and thematically analysed. The thematic areas that emerged were carefully identified and the frequency of each theme was recorded. These were used to complement findings from the qualitative data in the form of vignettes and direct quotes.

### Ethical considerations

The Research Ethics Board of the University of Ghana School of Graduate Studies approved this research protocol. All household survey questionnaires and individual interview schedules were administered with individual verbal informed consent. Each respondent was given a detailed explanation regarding the study objectives and procedures. Study participation was voluntary and each respondent was given an opportunity to withdraw from the study at will. Confidentiality was maintained by making the data accessible to only the members of the research team.

## Results

### Socio-demographic characteristics of respondents

There were slightly more females (53.4%) than males (46.6%) in the sample. A little over three quarters (78%) of the respondents were aged between 30 and 49 years. The age of the respondents ranged from 20 to 79 years old. With regard to level of education, 27% of the sampled respondents had primary education, 62% had middle or junior high education, and 11% have had secondary (senior high) education and above. Three percent of the respondents are currently enrolled as students. The majority (78%) of the respondents indicated they were self-employed, mostly as retailers and small-scale farmers. About 6% have retired. A little over half (51%) of the respondents were identified as Ga-Adangbe. Most of the respondents (90%) were affiliated with the Christian religion. These results are presented in Table 1.

**Table 1:** Socio-demographic characteristics of respondents

Variable	Frequency	Percent (%)
Gender		
Male	157	47
Female	180	53
Age		
20–29	16	5
30–39	184	55
40–49	80	24
50–59	36	11
60–69	14	4
70–79	7	2
Education		
Primary	91	27
Middle/junior high	207	61
Secondary	25	7
Tertiary	14	4
Employment status		
Employed	38	11
Self-employed	262	78
Student	9	3
Unemployed	7	2
Retired worker	21	6
Ethnicity		
Akan	46	14
Ewe	100	30
Ga-Adangbe	171	51
Guan	14	4
Mole-Dagbani	6	2
Religious affiliation		
Christian	303	90
Muslim	28	8
Traditionalist	6	2
Total	337	100

### Knowledge of malaria

Respondents were made to state what they recognize to be the cause of malaria as well as the major symptoms associated with malaria illness. The results show that almost all the respondents (99%) recognize infected mosquito bite as the cause of malaria with only 1% indicating overworking as a cause of malaria. Some nuances are observed when stratified by educational attainment of the respondents. All respondents (100%) who had at least secondary level of education correctly attributed the cause of malaria to the bite of an infected mosquito. However, about 4% of respondents with less than secondary education as their highest educational attainment attributed the cause of malaria to overworking. Bivariate tests were performed to access whether individuals’ socio-demographic characteristics associated significantly with their knowledge about the cause of malaria. Results from the bivariate analyses are presented in Table 2. The results indicated that individuals’ religious affiliation was the only variable that significantly associated with their knowledge about the cause of malaria (*p* value < 0.001). While only a minuscule of respondents who identified with the Christian religion (0.3%) incorrectly associated the cause of malaria with working in the sun, 7% of respondents who were affiliated with other religions (Muslim and Traditional African Religions) incorrectly attributed the cause of malaria to working in the sun.

**Table 2:** Association between respondent socio-demographic characteristics and knowledge about malaria

	What do you think causes malaria?		Yates’ Chi square
	The bite of an infected mosquito	Overworking in the sun	
Gender			2.680
Male	154 [97%]	4 [3%]	
Female	179 [100%]	0 [0%]	
Age			0.094
20–49	271 [99%]	4 [1%]	
50–79	62 [100%]	0 [0%]	
Education			0.006
Primary/middle/junior high	295 [99%]	4 [1%]	
Secondary/tertiary	38 [100%]	0 [0%]	
Religion			12.731*
Christian	303 [99.7%]	1 [0.3%]	
Other	30 [91%]	3 [7%]	
Ethnicity			0.162
Ga-Adangbe	174 [98%]	3 [2%]	
Other	159 [99%]	1 [1%]	

* Yates’ p value < 0.001

**Fig. 2:**
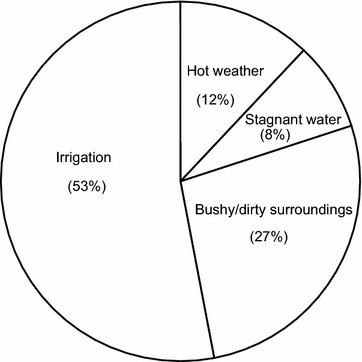
Perceived factors promoting mosquito breeding

Regarding the natural and social conditions that promote the breeding of mosquitoes, respondents perceive stagnant water bodies, bushy and dirty environment, irrigation, and hot weather as the proximate factors contributing to the breeding of mosquitoes in the area. A little over half (53%) recognize irrigation as the most immediate factor promoting the breeding of mosquitoes in the area (Fig. 2).

Responses in the qualitative interviews on the factors that promote the breeding of mosquito in Asutsuare support findings from the quantitative data above. The following quotations point to the fact that irrigation is recognized as the major factor that promotes the breeding of mosquitoes in the area:

*“The major factor is the rice farms because a lot of stagnant water remains in the field when the canal is opened. If you go to the farm around 5:30* *a.m. or from 5:30* *p.m. going, a lot of mosquitoes will be all over your body and will be biting you”. (A leader of farmers).*

*“Mosquitoes are plenty here. The canal water which is used for the rice farms is the main reason”. (An opinion leader).*

*“Due to the rice farms and the canal, there is plenty mosquito around”. (An elder of the Chief’s Palace)*

When asked to indicate what they recognize to be the major symptoms of malaria illness, a quarter (25%) of the respondents stated severe headache, followed by bodily weakness (20%), and dizziness (18%). The remaining 37% of the respondents listed other symptoms including fever and loss of appetite. Overall, it was noticed that the respondents were very knowledgeable about some of the most common clinical symptoms of malaria as outlined in the works of [21], and more recently [22].

Report from the qualitative interviews also show similar findings, where some respondents provided information on the usual symptoms people complain about, which make them suspect they have malaria. In the words of a key informant who works as a local drug store attendant:

*“The people I attend to usually complain of severe headaches, dizziness and bodily aches” (A drug store attendant).*

The following direct quotes from other key informants show similar symptoms:

*“Whenever I interact with the farmers, many of them complain of headaches, bodily pains, bitter mouth, dizziness* etc*.” (A field officer of the K.I.P).*

*“Reports from the clinic show that malaria is the commonest illness that is attended to. People usually complain of headaches, bodily pains, dizziness* etc*.” (An opinion leader)*

The generally high awareness of the cause and symptoms of malaria among the study participants is consistent with the results from earlier studies showing that awareness of malaria is generally high in high risk communities [23, 24].

### Practices towards malaria

As part of the objectives of this study, there was an assessment of the coping strategies adopted by the people in Asutsuare to treat and also prevent malaria infection. As to whether or not one would ever consider visiting a health facility for the treatment of malaria, all the respondents (100%) answered in the affirmative. However, only 3% of the respondents indicated they immediately visit a healthcare facility whenever they suspected malaria illness. The overwhelming majority (about 93%) would consider visiting a professional healthcare facility only when they themselves or family members have tried other options for treating the malaria infection and have failed. These respondents, without first testing for malaria, obtained medication (anti-malarial tablets and pain killers) from local drug stores (kiosks) and also herbal preparations from traditional healers, rather than visiting a formal healthcare facility where proper diagnostic tests are available.

This high prevalence in home or self-medication was highlighted during the qualitative interviews. The following quotations are illustrative:

*“When I go round the fields in the mornings, I see a lot of the farmers taking pain killers. You can even find the packets of drugs like “diclo” and “efpac” on the ground. If they take the pain killer once in a while that’s fine, but you see them always taking pain killers and I think it is not right”. (A field officer of the K.I.P).*

*“There are some people who start the first dose of anti*-*malarial drugs at home before coming here. Some even go up to 2nd or 3rd dose or complete their course. About 70% start treatment at home before coming to the clinic. They buy the drugs from the shop or drug peddlers because they want to avoid long queues at the clinic. Then when the drugs don’t work they come here. Those who live afar are the ones who normally try the drug peddlers first before coming to the clinic and they are those who normally come with severe malaria” (A health officer at the Osudoku Health Centre).*

Factors such as time constraints particularly among the farmers and long distances to the health facility were frequently cited for the high prevalence in home or self-medication. The following quotation from a community leader below captures this situation:

*“People don’t go to the clinic because they are farmers and they think attending hospital will waste their time and affect their farming. They will rather go to the drug store or prepare some herbs. They only go to the hospital when their condition is very serious”. (An opinion leader).*

The above statements suggest that, residents of the area mostly try to manage health conditions suspected to be malaria their own way so that until one’s own method for treating the disease has failed, visiting the health facility for the treatment of malaria is not an option for most people. This situation is quite typical for most rural areas in developing countries. A study by [25] in Oyo State, Nigeria, made a very similar observation, where it was reported that most people visited formal health care facilities such as clinics or hospital for health care only when home management practices have failed.

Further analysis on the form of support for the control and prevention of malaria in the area revealed interesting results. In all, 214 respondents (64%) stated that they had received support from both governmental and non-governmental organizations in the form of subsidized or free long-lasting insecticide-treated bed nets (LLINs). However, only 33 respondents (15%) out of the 214 who had benefitted from LLINs distribution programs and duly owned LLINs indicated they actually sleep in the nets at night. The following quotations from the interviews shed some light on the misuse of LLINs as well as some of the challenges that discourage their use:

*“We get free mosquito nets from the health people once in a while but not everyone gets. Even those who get the nets, some of them don’t use it. Some will rather use the net to fence their gardens” (An opinion leader).*

*“Sometimes we get mosquito nets but not everybody gets it. Many people say sleeping inside the net is hot so they don’t use it” (a leader of farmers).*

The above results suggest that unwillingness to use LLINs could also be a reality even among people living in high-risk malaria areas.

## Discussion

Malaria is endemic and perennial across Ghana. Over the past couple of decades, the ecological impacts of human activities such as the construction of irrigations systems and dams have received research attention as a contributing factor for the malaria menace in the country. This study sought to understand how local inhabitants in an irrigated community in rural Ghana perceived about the aetiology of malaria, as well as their attitudes towards proven preventive and treatment options.

Results from this study showed that education was an important factor that associated with the respondents’ perception about the causes of malaria. While respondents who have completed at least secondary school correctly attributed malaria to the bite of an infected mosquito, some respondents with no or up to junior high level of education incorrectly attributed malaria to overworking as well as working in the sun. The findings of this study support previous study in Kenya where [23] reported that, people with formal education were significantly more likely to associate mosquitoes with malaria than those who had no formal education [24] also made similar findings where they studied the relationship between malaria and agriculture in the Mvomero District in Tanzania. In their study, they found that respondents with at least primary school education were more knowledgeable on malaria related issues than those without formal education.

The study has reiterated the relevance of individuals’ socio-economic and demographic characteristics in the management of malaria. Individuals’ educational attainment and religious affiliations emerged as particularly important factors for understanding the aetiology of malaria. As regards education, we observed that all individuals with at least secondary level of education correctly attributed the cause of malaria to an infected mosquito. It is therefore very plausible that governmental interventions to increase access to secondary education, particularly for poor and rural residents, could have an additional effect of improving the overall health outcomes of the population.

The relevance of religion as a factor that is significantly associated with the knowledge about the aetiology of malaria, as observed in our bivariate analysis, warrants some attention. Within the Ghanaian context where religion plays an important part in people’s daily lives [26–28], sending malaria campaign messages to religious settings could be an effective approach to reach a large population especially when the information is being relayed by their religious leaders.

Overall, however, our findings indicate a generally high level of awareness among our research respondents from a rural agricultural setting about the aetiology of malaria as well as the identification of some clinical symptoms commonly associated with malaria illness. A little over half of the respondents also attributed the problem of malaria in the area directly to the presence of the irrigation projects. This level of awareness is similar to what earlier studies conducted in urban centres in Ghana showed (see, e.g., [9, 14]). While the high awareness of malaria, particularly in a rural endemic area is important, this does not necessarily translate into the recommended treatment and prevention efforts among most of the respondents.

The study observed that majority of the respondents subjectively judge the severity or otherwise of a suspected malaria illness before they decide to seek treatment from a professional healthcare personnel. Thus, until all self-medication options have failed and/or the condition of the sick person becomes severe (in their own subjective evaluation of what severity means), one may not see the need to visit a formal healthcare facility for suspected malaria treatment. Such delays in seeking professional treatment could result in further complications, poor case monitoring and surveillance systems as well as drug resistance to the malaria parasite [1].

This is particularly true in situations where people generally self-medicate on the basis of the symptoms they experience. Thus, individuals who experience headaches or bodily aches (a typical symptom of malaria illness) often buy local analgesics or painkillers as the first course of treatment without testing for malaria. This in fact, was an observation of a key informant who works as a drug store attendant in our study area:

*“Most of the people who come to me buy pain killers for a long time before realizing they need to treat malaria” (a drug store operator).*

Since malaria testing kits are not available in the study area, it is difficult for a drug dispensing personnel to provide an appropriate first-line malaria treatment. It is recommended that there must be malaria testing training for drug dispensing personnel as well as the provision of malaria testing kits in drug dispensing stores especially in rural endemic areas in order to facilitate early malaria screening and timely diagnosis.

The malaria control campaign needs to include key strategies for the early detection of malaria and also promote key messages about the timely reporting of suspected malaria illnesses in order to realize the goal of the National Malaria Control Programme of Ghana at reducing the country’s malaria morbidity and mortality by 70% by the year 2020 (compared with 2012 levels) via, inter alia, the immediate and universal diagnosis of all suspected malaria cases [11].

The results also suggested that many of the respondents could be unwilling to adopt the use of proven malaria prevention strategies such as the LLINs. Like several other rural endemic areas of Ghana, some study participants also reported being recipients of LLINs from either governmental or non-governmental agencies as part of the national malaria control programme. Consistent with results from the nationally representative population-based demographic and health survey [29], the results from this study showed that majority of those who claimed to have ever received LLINs as a form of support for malaria management and control, and therefore owned LLINs, may not actually be sleeping under them.

The findings are similar to those of earlier studies such as that of [30] in which they showed that many people who owned LLINs in Ghana do not actually use them due to several household constraints such as the unsuitability for floor mats, which are commonly used for sleeping in rural areas. This non-use of LLINs could gravely undermine the crucial anti-malaria efforts being carried out by relevant health agencies in the country. Institutions concerned with malaria control in rural Ghana must, therefore, focus more on strengthening the educational programmes targeted at the management and control of the malaria, with particular focus on strategies to promote preventive strategies like the use of LLINs in rural Ghana. In addition to the promotion of LLIN use, it will also be worthwhile to invest into other malaria preventive efforts such as a scheduled larvicide programme in endemic rural areas.
